# Sustained neurosurgical decision support through remote multidisciplinary case conferencing: A six-year LMIC-HIC collaboration

**DOI:** 10.1016/j.bas.2026.107473

**Published:** 2026-10-12

**Authors:** Rupavathana Mahesparan, B. Dhuvarahan, S.P. Athiththan

**Affiliations:** a Department of Neurosurgery, Haukeland University Hospital, Bergen, Norway; b Department of Clinical Institute 1, Faculty of Medicine, University of Bergen, Norway; c Medical Museum and Telemedicine Unit, Teaching Hospital Jaffna, Sri Lanka; d Teaching Hospital Jaffna, Sri Lanka

**Keywords:** Global neurosurgery, Remote multidisciplinary case conferencing, Telemedicine, Low- and middle-income countries, Teleradiology, Neurosurgical decision support, Implementation, Health systems strengthening

## Abstract

**Introduction:**

Global disparities in neurosurgical care remain substantial in low- and middle-income countries (LMICs), where limited access to specialist expertise and supporting services can restrict timely treatment. Remote multidisciplinary case conferencing may provide specialist decision support using existing digital infrastructure, but longitudinal descriptions remain limited.

**Research question:**

Can a low-cost, clinician-led remote case-conference model provide sustained neurosurgical decision support in a resource-constrained setting?

**Methods:**

We conducted a descriptive implementation case study of a collaboration between Haukeland University Hospital, Bergen, and Teaching Hospital Jaffna, Sri Lanka, from September 2019 to December 2025. Routine meeting logs were reviewed for meeting activity, case-discussion volume, imaging review, participants, workflow, and decision-support functions. The model combined asynchronous review of imaging and clinical information with synchronous multidisciplinary videoconferencing; direct remote patient examination was not routine.

**Results:**

Across 150 meetings, 1258 case-discussion episodes were recorded, involving review of more than 2700 CT and MRI studies. Some patients were discussed more than once, and the number of unique patients could not be reliably reconstructed. Discussions addressed diagnostic clarification, operative and non-operative management, surgical prioritization, additional investigation, follow-up, and referral or escalation. The model used existing digital infrastructure and professional time without dedicated project funding. Final clinical responsibility remained with the Sri Lankan treating team.

**Conclusion:**

This cross-border model was maintained for more than six years and demonstrates the feasibility of longitudinal neurosurgical decision support in a resource-constrained setting. Effects on clinical outcomes, professional development, implementation sustainability, and local capacity require prospective evaluation.

## Highlights

• Describes a six-year, low-cost, clinician-led neurosurgical telemedicine collaboration between Norway and Sri Lanka, focused on sustainable capacity building.
• Clarifies the practical workflow, including asynchronous teleradiology review, synchronous multidisciplinary discussion, documentation boundaries, and locally retained clinical responsibility.
• Reports longitudinal activity data from 150 multidisciplinary meetings, 1258 patient case discussions, and more than 2700 CT/MRI studies.
• Provides a pragmatic framework including workflow structure, cost description, governance considerations, and replicable strengths for LMIC–HIC neurosurgical partnerships.
• Demonstrates how sustained remote specialist support can strengthen diagnostic decision-making, surgical prioritization, multidisciplinary learning, local autonomy, and long-term neurosurgical capacity building.

## 1 Introduction

Neurosurgical services in many LMICs are constrained by workforce shortages, limited infrastructure, and restricted access to advanced imaging and perioperative support. These constraints affect access to safe and timely management of traumatic brain injury, hydrocephalus, brain and spinal tumors, vascular disease, and congenital neurosurgical conditions (Park et al., 2016; Dewan et al., 2019; Meara et al., 2015).

Sri Lanka has developed an established neurosurgical training pathway and several neurosurgical centers, but specialist density and distribution remain limited. A 2019 review reported 14 neurosurgical centers and 27 neurosurgeons in Sri Lanka, corresponding to a substantial workforce deficit relative to population need (Kato et al., 2020). Neurosurgical resources are also unevenly distributed, and regional referral hospitals may have limited access to subspecialty opinions, advanced equipment, intensive care beds, or timely oncological pathways.

Teaching Hospital Jaffna is the principal tertiary referral hospital for the Northern Province of Sri Lanka. During the period in which the collaboration was established, regional neurosurgical care depended on limited specialist personnel and patients could face substantial practical barriers to accessing subspecialty opinions elsewhere. Local decision-making also had to account for constraints in imaging availability, operative equipment, intensive care capacity, pathology and oncology pathways, and patient travel resources. These site-specific challenges created a need for rapid access to additional specialist discussion while preserving local clinical authority. The collaboration was therefore designed as advisory support rather than remote substitution for the local neurosurgical service.

Motivated by a commitment to global neurosurgical equity and a personal connection to the region, the first author initiated remote support for Teaching Hospital Jaffna in 2019. What began as informal professional contact evolved into regular cross-border multidisciplinary case conferencing that combined imaging review, neurosurgical consultation, and access to additional specialist perspectives when required. This article describes the operational model, scale, costs, governance, limitations, and lessons learned. The primary aim is to describe a longitudinal implementation experience and identify practical elements that may inform similar LMIC-HIC partnerships.

## 2 Methods

### 2.1 Study design and setting

This was a descriptive implementation case study based on routine program records from September 2019 to December 2025. The collaboration linked Haukeland University Hospital, Bergen, Norway, with Teaching Hospital Jaffna, Sri Lanka. It was established for clinical service support and professional collaboration rather than as a controlled trial or prospectively designed implementation study. Accordingly, the present analysis focuses on program continuity, activity, workflow, governance, and decision-support functions; implementation sustainability, educational outcomes, and capacity building were not prospectively measured as formal outcomes.

### 2.2 Remote case-conference model and platform

Meetings were conducted using Zoom and existing local digital infrastructure. The model was not a virtual outpatient clinic and did not include routine direct remote video examination of patients by the Norwegian neurosurgeon. It is therefore best described as remote multidisciplinary case conferencing supported by teleconsultation and teleradiology. Asynchronous review refers to review of available imaging and clinical summaries before or outside the live conference; synchronous review refers to real-time image review and multidisciplinary discussion during the videoconference.

Meetings were scheduled on Thursdays at 06:30 Central European Time when possible and were held weekly or biweekly depending on case volume and participant availability. Each session usually lasted 60-90 min. Cases were selected by the Jaffna neurosurgeon according to diagnostic uncertainty, complexity, urgency, limited local subspecialty access, or need for multidisciplinary input. The local team prepared relevant CT/MRI studies and concise clinical information before the meeting. Images were reviewed by screen sharing during the meeting and, when feasible, asynchronously before the discussion. The clinical intervention was the case-based specialist discussion and advisory decision support; the implementation strategy consisted of a stable local clinical lead, a consistent remote specialist relationship, a local telemedicine coordinator, recurrent meeting times, preparation of imaging and clinical summaries, use of existing digital infrastructure, and invitation of additional specialists when required.

### 2.3 Participants and evolution of the meetings

The core participants included the Jaffna neurosurgeon, the Bergen neurosurgeon, local medical officers attached to the neurosurgical unit, the teleradiology coordinator, and the supporting team involved in the collaborative process. Depending on the case mix, Sri Lankan radiologists, oncologists, pediatric neurologists, endocrinologists, and academic surgical colleagues participated. Additional international specialists were invited for selected cases where subspecialty input was needed. Junior doctors and medical students also attended several meetings. The Jaffna telemedicine coordination function supported scheduling, communication between participants, preparation and availability of imaging, and videoconference logistics. Over time, the meetings evolved from informal one-to-one support into regular multidisciplinary sessions. Historical logs did not consistently record attendance, cancelled sessions, connectivity failures, onboarding, or personnel turnover, and these implementation variables could therefore not be quantified retrospectively.

### 2.4 Case review workflow

The practical workflow is summarized in Fig. 1. Cases were identified and selected by the local treating team; relevant clinical information and imaging were prepared; asynchronous review was performed when feasible; and cases were then discussed synchronously in the multidisciplinary conference. Discussions focused on diagnosis, differential diagnosis, feasibility of local surgery, timing, preoperative preparation, need for additional imaging, oncological or endocrine input, conservative management, postoperative follow-up, and indications for transfer or escalation. Recommendations were explicitly adapted to local resource constraints, including availability of MRI sequences, operating microscope and instruments, blood products, intensive care beds, histopathology, radiotherapy, and patient travel limitations. Final management decisions and documentation remained with the Jaffna team. Re-discussion could occur after surgery, interval imaging, histopathology, or clinical change.

**Fig. 1:**
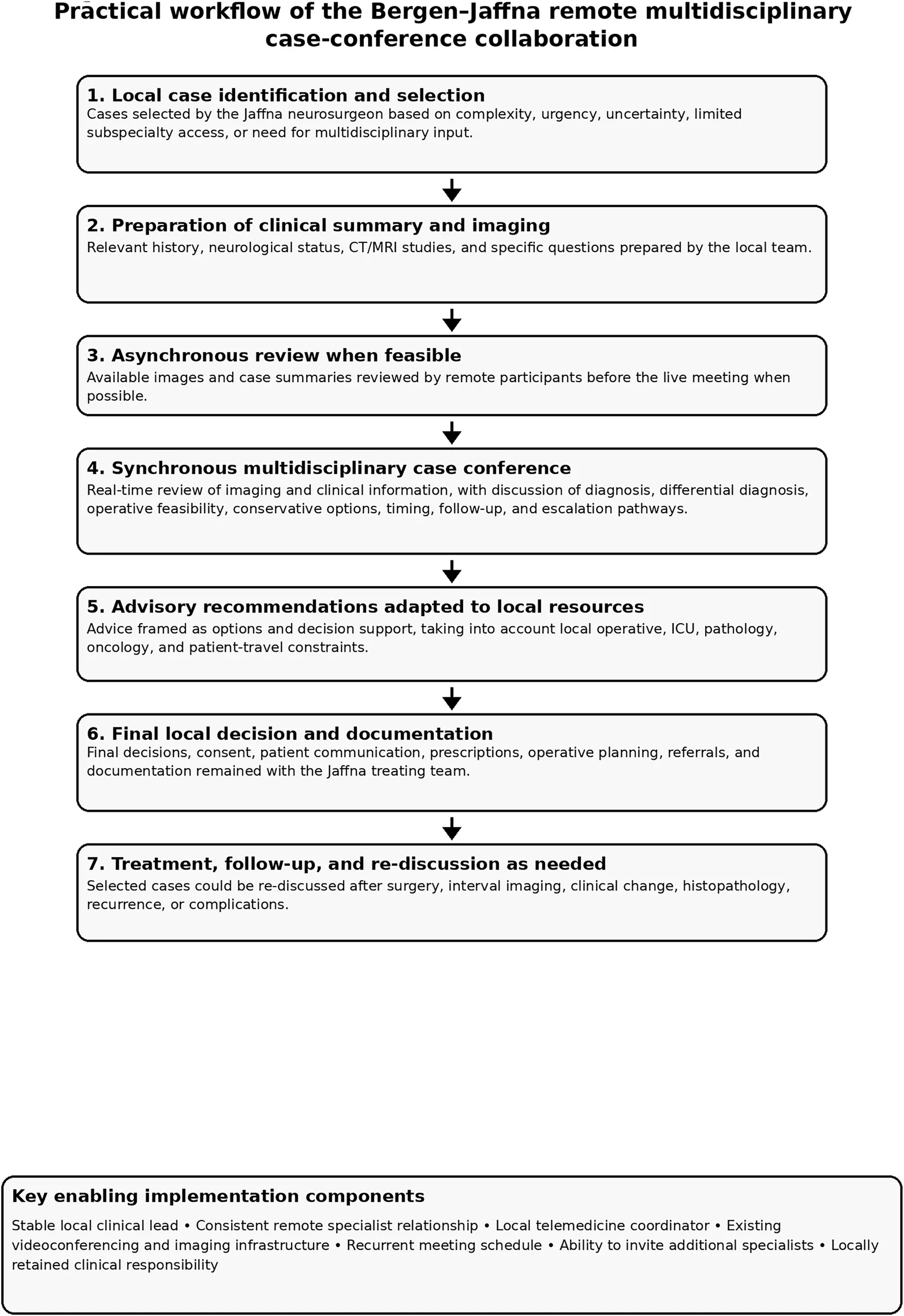
Practical workflow of the Bergen–Jaffna remote multidisciplinary case-conference collaboration. Remote specialist input supported local decision-making, while final clinical responsibility remained with the Jaffna treating team.

### 2.5 Data collection and evaluation

Routine meeting logs were used to summarize the number of meetings, case-discussion episodes, annual activity, and number of CT/MRI studies reviewed. The principal unit of observation was a case-discussion episode rather than a unique patient. Individual patients could be discussed on more than one occasion, for example after surgery, new imaging, histopathological clarification, or clinical change. Historical records did not permit reliable identification of unique patients throughout the complete study period; consequently, the reported 1258 discussions should not be interpreted as 1258 unique patients. Because the collaboration was initially established for clinical support rather than research, early records did not consistently capture detailed patient-level outcomes, transfer decisions, diagnostic categories, attendance, or whether a case represented isolated imaging review versus a full clinical discussion. We therefore report the aggregated activity variables that were consistently available and describe case mix and recurrent decision-support functions qualitatively. Variables not systematically recorded are treated as unavailable for retrospective analysis rather than as research missing data. This limitation informed the proposed prospective data framework presented later in the manuscript.

### 2.6 Governance, documentation, and medicolegal boundaries

The collaboration was structured as advisory decision support. The remote team did not prescribe treatment, order investigations, obtain consent, or assume direct clinical responsibility. Final decisions, communication with patients and families, consent, operative planning, and documentation remained with the Jaffna treating team. The Jaffna team documented relevant conclusions in the local medical record according to local policy. Patient-identifying information was minimized in cross-border communication whenever possible, and images were reviewed through screen sharing or local systems rather than creating a separate Norwegian clinical record. These boundaries shaped the type of recommendations considered feasible: the remote team could provide diagnostic interpretation, risk discussion, and management options, but recommendations had to remain advisory and locally actionable.

### 2.7 Cost description

The program was designed as a low-cost model using existing computers, internet access, local imaging systems, and an established videoconferencing platform. No dedicated project grant or separate reimbursement was provided for this neurosurgical collaboration. The main costs were clinician and coordinator time, preparation time, communication, and associated opportunity costs in addition to usual duties. The videoconferencing arrangement used existing local or institutional infrastructure rather than a platform purchased specifically for the project. A structured cost summary is provided in Table 1.

**Table 1:** Cost and resource components of the telemedicine model.

Resource component	How it was addressed	Implication for sustainability
Digital platform	Existing videoconferencing (ZOOM) and screen-sharing tools were used.	No need for dedicated commercial telemedicine infrastructure.
Imaging access	Local CT/MRI studies were displayed through local systems or screen sharing.	Avoided creating a parallel foreign clinical image archive.
Coordination	The local telemedicine coordinator and neurosurgeon prepared meetings and cases.	Coordination time became the main operational requirement as activity increased.
Clinical expertise	Local and international specialists contributed through voluntary professional time.	Low direct cost but vulnerable to competing duties and changes in individual availability.
Documentation	Clinical decisions were documented locally by the treating team.	Maintained local responsibility and reduced medicolegal ambiguity.

## 3 Results

### 3.1 Program scale

Between September 2019 and December 2025, 150 multidisciplinary meetings were conducted. During these meetings, 1258 case-discussion episodes were recorded, and more than 2700 CT and MRI studies were reviewed. Annual case-discussion activity was 3 episodes during the September-December 2019 start-up period, followed by 190 in 2020, 165 in 2021, 208 in 2022, 206 in 2023, 196 in 2024, and 290 in 2025 (Table 2 and Fig. 2). These raw annual counts reflect different calendar exposure in the start-up year and potentially varying meeting opportunities; they are therefore presented descriptively and are not interpreted as measures of adoption, program reliance, or maturation. The historical dataset did not consistently capture the number of meetings by year in a form suitable for exposure-normalized analysis.

**Table 2:** Annual case volume in the Bergen-Jaffna telemedicine collaboration, 2019-2025.

Year	Cases reviewed and discussed	Comment
2019	3	Start-up phase
2020	190	Regular case review established
2021	165	Sustained activity during pandemic-related constraints
2022	208	Expanded multidisciplinary participation
2023	206	Stable weekly/biweekly model
2024	196	Continued MDT use
2025	290	Highest annual activity
Total	1258	More than 2700 CT/MRI studies reviewed

**Fig. 2:**
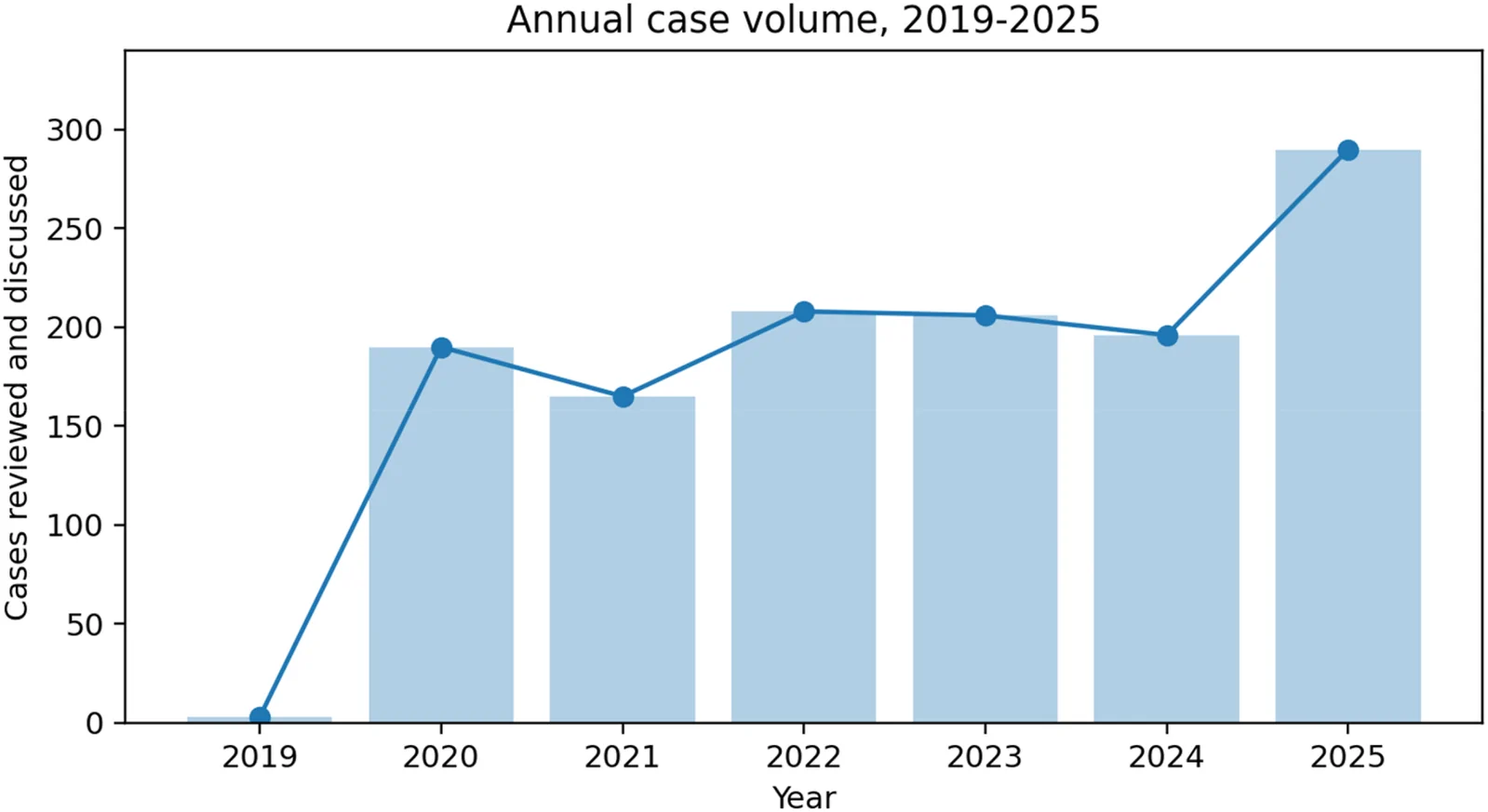
Annual case-discussion activity in the Bergen–Jaffna collaboration, 2019–2025.

### 3.2 Case mix and decision-support outputs

The available logs indicate a broad case mix. Discussions commonly involved neuro-oncology, including intra-axial tumors, pediatric brain tumors, meningiomas, pituitary adenomas, and vestibular schwannomas. Pediatric neurosurgical conditions, including hydrocephalus and congenital malformations, were also reviewed, as were selected spinal, vascular, trauma, infection, hemorrhage, and acute hydrocephalus cases. Because diagnostic categorization was not recorded consistently across the full study period, these descriptions are qualitative and should not be interpreted as complete category counts or proportions. Recurrent decision-support functions are summarized in Table 3.

**Table 3:** Recurrent decision-support outputs.

Decision-support domain	Typical contribution of the telemedicine meeting
Diagnostic clarification	Differentiating tumor subtype or radiological mimic; clarifying hydrocephalus versus ventriculomegaly; identifying cases suitable for follow-up rather than intervention.
Surgical prioritization	Identifying urgent cases, cases suitable for planned surgery, and cases where surgery should be deferred because expected benefit was low or perioperative risk was high.
Local management	Supporting locally feasible operative planning and postoperative follow-up
Transfer/escalation discussion	Considering referral or transfer when subspecialty treatment, oncology, or equipment needs exceeded local resources.
Follow-up strategy	Individualizing imaging intervals, re-discussion after clinical change, postoperative review, rehabilitation planning, and oncology/endocrine follow-up.

### 3.3 Illustrative case examples

The following de-identified examples illustrate the type of decision support provided. They intend to demonstrate the workflow and should not be interpreted as formal outcome measures.

• Complex brain tumor: A patient with a suspected intracranial tumor was reviewed to clarify imaging features, likely differential diagnosis, need for further imaging, need for biopsy, surgical approach, expected resectability, perioperative risk, patient-specific factors that could modify management, and the need for oncological input. The discussion helped the local team plan a feasible operative or non-operative pathway within available resources.
• Pediatric hydrocephalus/congenital case: Imaging and clinical history were reviewed to distinguish cases requiring urgent CSF diversion from cases suitable for observation, repeat imaging, or outpatient follow-up. Discussions focused on ventricular size, signs of raised intracranial pressure, associated congenital anomalies, timing of intervention, and feasibility of local treatment versus referral.
• Pituitary/skull base case: Endocrine, visual, and imaging considerations were discussed to determine whether local surgery, medical or endocrine work-up, delayed intervention, medical treatment, or referral for subspecialty treatment was most appropriate.
• Acute neurosurgical cases: Head injury, intracerebral hemorrhage, spinal trauma, infections, and acute hydrocephalus were discussed to support decisions about emergency surgery, conservative management, timing of intervention, intensive care treatment, appropriate medical/antimicrobial treatment, and follow-up imaging.
• Postoperative and follow-up cases: Selected patients were reviewed after surgery to assess postoperative imaging, residual disease, complications, recurrence, rehabilitation needs, and indications for further surgery or non-operative management.

### 3.4 Educational opportunities and potential system contribution

Regular case-based discussions provided opportunities for continuing professional development and multidisciplinary learning. The educational content was practical and clinically oriented, focusing on imaging interpretation, surgical indications, operative planning, perioperative risk assessment, conservative management options, follow-up strategies, and pathways for escalation or referral. However, clinician competence, diagnostic confidence, educational outcomes, and changes in multidisciplinary practice were not formally measured; these activities should therefore be interpreted as potential capacity-building mechanisms rather than demonstrated capacity-building outcomes.

With time, the meetings became more structured and functioned as a recurring multidisciplinary forum in addition to providing remote management advice. Junior doctors and medical students attended a number of meetings, extending the potential educational reach beyond the treating neurosurgeon. Attendance and educational participation were not systematically quantified across the full study period, and no causal inference regarding local capacity development can be made from the present data.

### 3.5 Costs and continuity

The collaboration was maintained for more than six years without dedicated external project funding. It depended on existing digital infrastructure, the Jaffna telemedicine coordination function, and professional time from local and remote specialists. As the program continued, coordination rather than new equipment remained the principal operational requirement. The absence of dedicated funding reduced direct program costs but also left the collaboration vulnerable to competing clinical duties, participant availability, and reliance on individual commitment. Longitudinal continuity is directly observed in this study; formal implementation sustainability, acceptability, fidelity, penetration, and maintenance were not prospectively measured.

## 4 Discussion

This six-year collaboration demonstrates that a low-cost remote multidisciplinary case-conference model can be maintained longitudinally to provide structured neurosurgical decision support in a resource-constrained setting. Its principal documented contribution is repeated access to specialist discussion around diagnosis, surgical prioritization, conservative management, follow-up, and referral options. Potential effects on clinical outcomes, professional learning, local autonomy, and health-system capacity were not formally measured and should be considered hypotheses for prospective evaluation rather than demonstrated outcomes.

### 4.1 Remote case conferencing, telemedicine, and teleradiology

The term telemedicine can imply direct virtual patient assessment. In this project, the routine model was more accurately a hybrid of teleradiology, teleconsultation, and remote multidisciplinary case conferencing. Direct video-based examination of patients was not performed as part of the routine workflow. The clinical reasoning process therefore resembled a conventional multidisciplinary team meeting, but differed in its cross-border structure, unequal access to subspecialty expertise, and explicit adaptation of recommendations to local resource constraints. The remote participants remained advisory and did not assume clinical responsibility; final decisions remained with the treating team at Teaching Hospital Jaffna. The first author also assessed patients in person during several visits to the collaborating institution during the project period, but these visits were complementary to, rather than part of, the routine remote workflow.

Future development of the collaboration could include structured video-based patient assessment in selected cases. Such an expansion would require clear procedures for patient consent, documentation standards, data protection agreements, interpreter support when needed, and clarification of medicolegal responsibilities.

### 4.2 Key strengths of the model

A major strength of this initiative was its longitudinal continuity. Meetings were maintained for more than six years without dedicated project funding or formal reimbursement, relying on professional commitment, a stable local clinical lead, a consistent remote specialist relationship, and existing digital infrastructure. This demonstrates operational feasibility and continuity but should not be interpreted as formal evidence of implementation sustainability as defined by implementation-science outcomes. The simplicity of the infrastructure may make the model relevant to other LMIC-HIC settings, although transferability and scalability require evaluation in additional contexts.

A second strength was that the collaboration preserved local clinical authority. Remote input provided diagnostic interpretation, surgical strategy, risk discussion, and management options, but did not replace the authority of the Sri Lankan treating team. All final clinical decisions, patient communication, consent, and documentation remained local. This structure was intended to keep recommendations realistic within available operative, ICU, oncology, pathology, and patient travel resources. The present study documents this governance model but does not measure changes in local autonomy or decision quality.

A third strength was the combination of practical clinical support with repeated opportunities for case-based education and multidisciplinary learning. This was relevant across neuro-oncology, pediatric neurosurgery, pituitary and skull base disease, trauma, infection, and spinal pathology. The first author also brought prior experience from neurosurgical education and capacity-building partnerships in LMIC settings, including long-standing engagement in Addis Ababa, Ethiopia. This background influenced an emphasis on mentorship and context-sensitive advice; however, the educational and capacity-building effects of the Jaffna collaboration itself were not formally assessed in the present study.

Finally, the collaboration sought to align established neurosurgical principles with local realities. Discussions emphasized safety, appropriateness of intervention, realistic operative goals, conservative alternatives, and responsible use of limited resources. In this respect, the model functioned as more than remote image review: it provided a structured forum for advisory clinical reasoning while preserving locally retained responsibility.

Why the model was feasible.

Several factors appeared to support feasibility: a clearly identified local clinical lead, a stable remote specialist relationship, a local telemedicine coordinator, recurrent meeting times, case selection by the treating team, use of existing technology, and respect for local autonomy. These elements represent the implementation strategy surrounding the clinical case-conference intervention. The model avoided dependence on expensive new platforms or repeated external missions. Whether the same strategy is acceptable, sustainable, or scalable in other settings requires prospective evaluation.

### 4.3 Comparison with literature

The findings are consistent with literature on global neurosurgery, telemedicine, and twinning-based partnerships, which emphasize continuity, mutual respect, local leadership, education, and context-sensitive decision-making (Eichberg et al., 2021; Punchak et al., 2023; Corell et al., 2024; Razak et al., 2025). The Lancet Commission on Global Surgery and global neurosurgery workforce studies have highlighted the importance of building surgical and neurosurgical capacity in resource-limited settings (Park et al., 2016; Dewan et al., 2019; Meara et al., 2015; Gupta et al., 2024). The present collaboration differs from many formal institutional programs because it began as an individual professional initiative and evolved into a recurrent clinical process. Its six-year continuity supports feasibility but should not be equated with formally measured sustainability or capacity-building outcomes.

The model also aligns with broader global neurosurgery initiatives emphasizing virtual collaboration, immersive technologies, and educational support (Lepard et al., 2020; Shlobin et al., 2023; Higginbotham, 2021; Ukachukwu et al., 2024; Rosseau et al., 2020; Garcia et al., 2023). Its distinct contribution is the description of an operationally simple, low-cost, longitudinal workflow that combines asynchronous image review with synchronous multidisciplinary discussion and local clinical responsibility. In the revised interpretation, the current study is best viewed as a descriptive implementation experience. Future evaluation should draw on established implementation and telemedicine frameworks, including StaRI, Proctor implementation outcomes, MAST, and SQUIRE, to measure adoption, fidelity, acceptability, maintenance, educational effects, and relevant clinical outcomes (Pinnock et al., 2017; Proctor et al., 2011; Kidholm et al., 2012; Ogrinc et al., 2016).

### 4.4 Medicolegal and ethical considerations

Cross-border clinical advice raises questions about documentation, liability, privacy, and responsibility. These concerns influenced project design. The remote specialists provided advice rather than direct care; recommendations were framed as options; patient-specific decisions were made and documented locally; and data sharing was minimized. For other programs, formal memoranda of understanding, agreed documentation templates, data protection procedures, and institutional recognition should be considered, particularly if direct patient video assessment, image transfer to foreign servers, or patient-level outcome research is introduced.

### 4.5 Limitations

This report has important limitations. It is a descriptive implementation case study without a control group, formal patient-reported outcomes, or systematic assessment of educational or implementation outcomes. The unit of observation is a case-discussion episode rather than a unique patient, and repeat discussions cannot be reliably separated across the full historical dataset. Routine logs provide robust aggregate activity data but did not consistently capture annual meeting opportunities, participant attendance, cancellations, connectivity failures, diagnostic categorization, treatment decisions, local management versus transfer, implementation of recommendations, operative outcomes, complications, or long-term follow-up. Consequently, raw annual case-discussion counts cannot be interpreted as uptake or reliance, and the reported clinical contribution reflects decision-support processes rather than measured causal effects on patient outcomes. The program also depended substantially on individual commitment, which may limit transferability. These limitations should be addressed through prospective structured data collection, stakeholder evaluation, and patient-level outcome tracking.

### 4.6 Future evaluation framework

Future phases should include prospective, structured data collection to document clinical, educational, and implementation outcomes. For each case, the type of review should be recorded, such as isolated imaging review, full clinical case discussion, postoperative follow-up, emergency decision support, or educational review. The dataset should distinguish unique patients from repeated case-discussion episodes and record meeting opportunities, attendance, cancellations, technical interruptions, and relevant adaptations. Recommended management should also be documented, including local surgery, conservative treatment, repeat imaging, referral, transfer, or outpatient follow-up. Implementation outcomes should include adoption, fidelity, acceptability, penetration, and maintenance, using established frameworks where appropriate.

Where feasible, follow-up should capture whether recommendations were implemented, modified, or deferred; time to surgery or specialist referral; complications; need for re-discussion; and available clinical outcomes. Structured feedback from participating clinicians, junior doctors, and medical students could assess educational effects, including diagnostic confidence, surgical planning, multidisciplinary collaboration, and complex decision-making. Measures of local decision-making without remote input, referral patterns, and stakeholder-reported value would help determine whether repeated case conferencing contributes to capacity development rather than merely providing continued external support.

## 5 Conclusion

This clinician-led collaboration between Bergen and Jaffna demonstrates that a low-cost, cross-border remote multidisciplinary case-conference model can be maintained for more than six years using existing technology and locally retained clinical responsibility. Across 150 meetings, 1258 case-discussion episodes and more than 2700 imaging studies were reviewed, providing repeated opportunities for diagnostic clarification, management discussion, surgical prioritization, follow-up planning, and referral decisions. These observations support operational feasibility and longitudinal continuity, but do not by themselves establish improved clinical outcomes, formal implementation sustainability, or neurosurgical capacity building. Prospective evaluation is needed to determine the model's clinical, educational, implementation, and transferability outcomes.

## Disclosure-conflict of interest statement

The authors declare that they have no financial or non-financial conflicts of interest related to this work. No dedicated funding was received for this telemedicine collaboration or for the preparation of this manuscript. The collaboration was conducted using existing institutional infrastructure and voluntary professional time. The authors alone are responsible for the content of the manuscript.
